# Criteria for assessing peripheral nerve injury. Behavioral and functional assessment in non-operated Wistar rats ^1^

**DOI:** 10.1590/s0102-865020200070000002

**Published:** 2020-08-17

**Authors:** Renan Kleber Costa Teixeira, Faustino Chaves Calvo, Deivid Ramos dos Santos, Nayara Pontes de Araújo, Daniela Ferreira Tramontin, Luís Vinícius Pires da Costa, Rui Sergio Monteiro de Barros

**Affiliations:** I MD, MS, Department of Experimental Surgery , School of Medicine , Universidade do Estado do Pará (UEPA), Belem - PA , Brazil . Conception, design, intellectual and scientific content of the study; interpretation of data; statistical analysis; critical revision.; II Graduate student, School of Medicine , UEPA , Belem - PA , Brazil . Acquisition and interpretation of data, manuscript preparation.; III Fellow Master degree, Postgraduate Program in Surgery and Experimental Research , UEPA , Belem - PA , Brazil . Acquisition and interpretation of data, manuscript writing.; IV Graduate student, School of Medicine , Universidade Federal do Pará (UFPA), Belem - PA , Brazil . Acquisition and interpretation of data, manuscript preparation.; V Graduate student, School of Medicine , UFPA , Belem - PA , Brazil . Acquisition and interpretation of data.; VI Graduate student, School of Medicine , UEPA , Belem - PA , Brazil . Acquisition and interpretation of data.; VII PhD, Associate Professor, Department of Experimental Surgery , School of Medicine , UEPA , Belem - PA , Brazil . Scientific content of the study, final revision.

**Keywords:** Peripheral Nerve Injuries, Behavioral Research, Behavior Rating Scale, Rats, Introduction

## Abstract

**Purpose:**

To evaluate the normality pattern in functional tests of peripheral nerves.

**Methods:**

Sixty female and sixty male Wistar rats were submitted to vibrissae movement and nictitating reflex for facial nerve; grooming test and grasping test for brachial plexus; and walking tracking test and horizontal ladder test for lumbar plexus. The tests were performed separately, with an interval of seven days between each.

**Results:**

All animals showed the best score in vibrissae movement, nictitating reflex, grooming test, and horizontal ladder test. The best score was acquired for the first time in more than 90% of animals. The mean of strength on the grasping test was 133.46±12.08g for the right and 121.74±8.73g for the left anterior paw. There was a difference between the right and left sides. There was no difference between the groups according to sex. There is no statistical difference comparing all functional indexes between sex, independent of the side analyzed. The peroneal functional index showed higher levels than the sciatic and tibial functional index on both sides and sex.

**Conclusions:**

The behavioral and functional assessment of peripheral nerve regeneration are low-cost, easy to perform, and reliable tests. However, they need to be performed by experienced researchers to avoid misinterpretation.

Peripheral nerve injuries are a common entity that is related to blunt or penetrating trauma ^1^ . Despite the neural regenerative potential, motor and/or sensory function is rarely restored spontaneously after an injury ^2^ . Disorders involving the peripheral nerves can have devastating impacts on patients’ daily functions and routines, quality of life and are associated with a high social cost due to early retirement and work limitations ^1 , 2^ .

Better magnification microscopes, advances in anatomic knowledge of peripheral nerves topography and a better understanding of the pathophysiology of nerve injury have all led to a decisive leap forward in the management of this condition ^3 , 4^ . Refined microsurgical techniques ^5 , 6^ such as neurorrhaphies (end-to-end, end-to-side, side-to-side, supercharging), neural neurotization, nerve transfer, nerves conduits, and others were developed; However, despite the encouraging results obtained when analyzing the technical, histological and electrophysiological parameters; these parameters normally do not correlate with the return of the functional recovery ^3 , 5 , 7 , 8^ , making the treatment of peripheral nerve injury a challenge to actual medicine.

In this context, several functional tests were developed to better study and understand the limiting factors in experimental studies. There are several functional tests described in the literature for the evaluation of different nerves or groups of nerves ^9 - 19^ . These tests do not evaluate reflex acts, but complex motor acts involving agonist and antagonist muscles ^11 , 13 , 16^ . And when there is an injury to a particular peripheral nerve, these quantify and qualify the degree of loss and allow to assess whether there was a functional recovery ^20 , 21^ .

Despite the studies describing these tests and their wide use in the literature, these were performed on animals with neural injury and without evaluation of the difficulties inherent in the tests. Thus, this study aims to evaluate the normality pattern in several functional tests of peripheral nerves.

## Methods

This research followed the rules of the Brazilian Law for Animal Care (Law: 11.794/08) and was approved by the Animal Use and Care Committee at Universidade do Estado do Pará.

Sixty female and sixty male Wistar rats ( *Rattus norvegicus* ) obtained from the Animal Colony of the Experimental Surgery Laboratory of UEPA were used. They were kept in a controlled environment with food and water *ad libitum* .

All animals were submitted to behavioral and functional assessment of peripheral nerve evaluated (vibrissae movement and nictitating reflex for facial nerve; grooming and grasping test for brachial plexus; and walking tracking test and horizontal ladder test for lumbar plexus). The tests were performed separately, with an interval of seven days between each. All tests were executed by just one person previously trained; and evaluated by two judges, except in grasping test. In case of divergence between the evaluators, a third evaluator independent reviews their decisions and makes the final choice by consensus.

Vibrissae movements ^9^ were scored on a 0–4 scale (0 - no movement; 1 - slight whisker movement; 2 - slow movement; 3 - rapid movement distinguishable from the contralateral normal side; and 4 - symmetric movement). The nictitating reflex ^10^ was scored on a 0–2 scale (0 - no reflex; 1 - nictitating reflex distinguishable from the contralateral side; 2 - nictitating reflex symmetric. To stimulate the nictitating reflex, a drop of saline 0.9% was instilled in each eye.

The grooming test ^11 , 12^ consisted of spraying water over the animals’ face to elicit grooming movements of the forepaws toward the head. In normal grooming, animals raised both forelimbs, licked them and reached up behind the ears. The grooming response was scored on a 0–5 scale (0 - no response; 1 - flexion at elbow, not reaching the snout; 2 - flexion reaching the snout; 3 - flexion reaching below the eyes; 4 - flexion reaching to the eyes; 5 - flexion reaching to the ears and beyond).

To perform the grasping test ^13 , 14^ , we used a lateralized grip strength meter manufactured by Bonther ^©^ (Ribeirão Preto – SP, Brazil). The rat is held around the abdomen and lowered into the apparatus so that it grasps the grip, with its back paws standing on the smooth floor of the apparatus. An experimenter holds the rat by the base of the tail and pulled it gently in a rearward direction, so the animals naturally cling to the grip to resist the pull. The applied force at which the rat releases the grip with each paw is measured separately. We performed three times the grasping test and used the highest result by each hand. The use of the opposite forepaw during the test was temporarily prevented by wrapping it round with adhesive tape.

The walking tracking test ^13 , 15 , 16^ was performed in a confined walkway with 8.7 × 43 cm track and with a dark shelter at the end. A white paper was cut to the appropriate dimensions and placed on the floor. The animals were held by the chest and their hind feet were pressed on an ink pad, and then, immediately, are allowed to walk along the track. At least, we used two paw prints by each side. The functional indices were calculated based on using the following parameters: 1- Print length (PL), distance from the heel to the third toe; 2- Toe spread (TS), distance from the first to the fifth toe; and 3- Intermediary toe spread (ITS), distance from the second to the fourth toe. The measurements are taken from the experimental (E) and normal (N) sides. The mathematical formula used for evaluation was described by Bain et al ^17^:

- Sciatic nerve: -38.3x[(EPL-NPL)/NPL] +109.5x [(ETS-NTS)/NTS] +13.3x[(EIT-NIT)/NIT] -8.8
- Tibial nerve: -37.3x[(EPL-NPL)/NPL] +104.4x[(ETSNTS)/ NTS] +45.6x[(EIT-NIT)/NIT] -8.8
- Peroneal nerve: 174.9x[(EPL-NPL)/NPL] +80.3x [(ETS-NTS)/NTS] -13.4

In horizontal ladder test ^13 , 18 - 20^ , skilled walking, limb placement and limb co-ordination were evaluated. It was realized with a horizontally positioned ladder, where the rats cross to escape an aversive stimulus (noise or light). The spacing between the rungs is variable and can be changed to prevent the animal from learning the position. The animals’ crossing was recorded to determine the number of foot fault scoring and forepaw digit score. The foot fault score was: 0 - Total miss, a fall occurred; 1 - The limb was initially placed on a rung, then slipped off and fall; 2 -The limb was placed on a rung, slipped off, but did not result in a fall nor interrupt the gait cycle; 3 - The limb was placed on a rung, but before it was weight-bearing it was quickly lifted and placed on another rung; 4 - The limb aimed for one rung, but was then placed on another rung without touching the first one, or a limb was placed on a rung and was quickly repositioned while remaining on the same rung; 5 - The limb was placed on a rung with either wrist or digits of the forelimb or heel or toes of the hindlimb; 6 - The midportion of the palm of a limb was placed on the rung with full weight support. The forepaw digit score was: 0 - Digits closed in an approximate 90° degree angle; 1 - Digits closed in an approximate 45° degree angle; and 2 - Digits completely flexed around the rung. Scores of five steps were averaged and used for analysis.

The parameters analyzed were weight (grams), sex (male *x* female) and the tests scores. The software BioEstat ^©^ 5.4 was used. All data were expressed as means ± standard deviation. The T student test was used to compare tests score according to laterality and weight, and the G test to compare tests score according to sex. Statistical significance was assumed at p< 0.05.

## Results

The weight of the animals was 327.83 ±27.55g. The weight of males was 340.22 ±30.81g and the female was 318.77 ±28.11g. There was no statistical difference between the groups (p=0.61). Table 1 resumes the score of all tests performed. All animals (male and female) show score 4 (best=4) bilaterally in vibrissae movement test. The best score was acquired for the first time in 36 males and 37 females, and the other animals got the grade 4 at second time. All animals (male and female) show score 2 (best=2) bilaterally in the nictitating reflex test. The best score was acquired for the first time in 39 males and 40 females, and others animals got grade 4 at the second time. There is no correlation between the weight or sex with the time required to perform the best score.

**Table 1:** Mean score of functional tests performed.

Tests performed/Sex		Male	Female
Vibrissae movement test		4.00 ± 0.00	4.00 ± 0.00
Nictitating reflex test		2.00 ± 0.00	2.00 ± 0.00
Grooming test		5.00 ± 0.00	5.00 ± 0.00
Grasping test ^*^	Right paw	136.11 ±10.84g	130.81 ±11.56g
	Left paw	123.44 ±10.22g	119.21 ±10.07g
Foot fault score		6.00 ± 0.00	6.00 ± 0.00
Forepaw digit score		2.00 ± 0.00	2.00 ± 0.00

^*^ p<0.05 (T student test) Right *vs* . Left paw in both sexes.

All animals (male and female) show score 5 (best=5) in the grooming test. The best score was acquired at the second time in 2 males and 3 females; in 7 males and 9 females at third time; in 24 males and 20 females; and in 7 males and 8 females at fourth time. There is no correlation between the weight, sex or the right and left side (p>0.05) with the time required to perform the best score.

The mean of strength on grasping test was 133.46 ±12.08g for the right anterior paw and 121.74 ±8.73g for the left anterior paw. In the male rats the mean was 136.11 ±10.84g for the right anterior paw and 123.44 ±10.22g for the left anterior paw; and in the female rats, the mean was 130.81 ±11.56g for the right anterior paw and 119.21 ±10.07g for the left anterior paw. There was no difference between the groups according to the sex (p=0.73); however, there was a correlation between the weight and the strength on grasping test (Pearson’s rho: 0.12, 95% IC: 0.04–0.40, p = 0.03). There was a difference between the right and left side (p=0.01), where the right side showed the greatest force.

**Figure 1:**
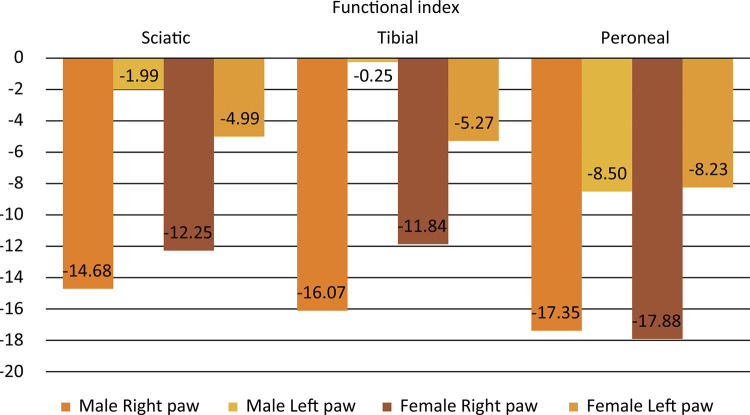
Mean of functional index tests performed according to sex and laterality.

Figure 1 resumes the results of the functional index tests performed. In the male rats, the sciatic functional index for the right posterior paw was -14.68 ±8.65 and for left posterior paw was -1.99 ±8.97; There was statistical difference between the sides (p=0.0004). The tibial functional index for the right posterior paw was -16.07 ±10.60 and for left posterior paw was -0.25 ±11.27; There was statistical difference between the sides (p=0.0002). The peroneal functional index for the right posterior paw was -17.35 ±10.05 and for left posterior paw was -8.50 ±10.34; There was statistical difference between the sides (p=0.0126).

In the female rats, the sciatic functional index for the right posterior paw was -12.25 ±9.72 and for left posterior paw was -4.99 ±5.22; There was statistical difference between the sides (p=0.0047). The tibial functional index for the right posterior paw was -11.84 ±8.29 and for left posterior paw was -5.27 ±5.76; There was statistical difference between the sides (p=0.0079). The peroneal functional index for the right posterior paw was -17.88 ±10.09 and for left posterior paw was -8.23 ±10.84; There was no statistical difference between the sides (p=0.1060).

There is no statistical difference comparing all functional index between male and female, independent of the side analyzed (p>0.05). There is no correlation between all functional index and weight (>0.05). The peroneal functional index shows the high levels than sciatic and tibial functional index in both sides and sex (p<0.05).

In horizontal ladder test, all animal scored 6 (best=6) in foot fault score and 2 (best=2) in forepaw digit score. The best score was acquired at the first time in all 80 animals; however, they needed to be training one week (in a different ladder) before performing the real test to evict “stops” or/and “return” of animals. There was no statistical difference between the sides (p>0.05) or gender (p>0.05).

## Discussion

The behavioral and functional assessment of peripheral nerve regeneration are simple tests that could score the grade of injury/recovery of motor function ^12 , 15 , 16^ . These assets are no invasive procedures, then could be applied several times different than histological and electrophysiological parameters ^20 , 21^ . Nonetheless, a researcher must be trained in how to do the test, before applying in research to avoid misinterpretation, as was identified in this paper where some normal animals need to execute more than three times one test to have the real score.

The vibrissae movements and nictitating reflex tests could be used alone or together to a better understanding of the nerve regeneration process, insofar as Haldock *et al* . ^23^ and Tomov *et al* . ^24^ studies described different and independent zones of facial function in rats. Although these tests have a “subjective assessment”; new studies are using software to evaluate with precision the vibrissae movements (whisking amplitude and velocity) and the blinks (analysis the maximum closed-angle and number of blinks per minute). A possibility for low-cost analysis is to perform a slow-motion record and search frame by frame the maximum whisking amplitude and/or the maximum eye closed angle and number of blinks per minute.

The grooming test surveys the C5/C6 roots and terminal branches of the rotator cuff. This test is a part of a normal stimulus, then in some animals, it is necessary to wait some minutes for the animal to perform the grooming. The absence of statistical difference between sex and weight allows the selection of better animals to the research that respect the particularity of the aim of the study ^24^ . Some studies highlight that some animals are right or left-handed ^26 , 27^ ; however, in this bigger sample the laterality doesn’t show an effect on the score or time required to perform the best score.

The grasping test analysis supports the right-handed of Wistar rats; this laterality is important in the selection of animals to avoid bias and backing the use of the right side of anterior paw in studies than the left side. Similar to grooming test, there is no effect of sex in the strength of grasping, allowing the use of female rats, which are normally avoided in studies ^28^ . The weight is a correlation with the age of young animals ^29^ , so we hypothesized that the correlation between the weight and strength on grasping is due to the difference in the age of animals; more studies analyzing this parameter are necessary to understand better this effect and its possible correlation with obesity and grasping of rats or/and hormonal effects on muscle.

The walking tracking test is a classic model to evaluate the sciatic and its branches function ^30^ , in this data the left side shows a better result than right side and the peroneal functional index than sciatic and tibial functional index. So, studies must use the left side as experimental and the right as control and evaluated the result using the peroneal functional index. We don’t know why happen this difference between the sides. In the literature review performed no studies compare the sides after a sciatic nerve injury. So, we hypothesized that the rats have a left-handed in posterior paw.

Although the most recent studies criticize this evaluation method, the main reasons are ^20 , 31 , 32^: 1) do not reflect maximal muscle force capacity; 2) do not differentiate models of motor dysfunction; 3) high time spent scoring animal behavior; and 4) consistency of data. Due to these factors, this test is being replaced by automatic (i.e. electric treadmills) tracking test and by ladders tests.

The horizontal ladder test is a dynamic kinematic analysis where many parameters could be assessed, such as ^18 - 20^: foot fault score, forepaw digit score, joint angles, walking pattern analysis and tracking test. It mimics the daily demands of living in an urban environment. Further, rung ladders are often used to enrich the cage environment for housing of laboratory rats ^20^ . This test is one of the most complete and complex behavioral and functional assessments; however, the training of animals is mandatory to avoid bias and animals’ anxiety ^19^ . This test could be adapted using an upward and/or downward rung ladder walking ^20^ , changing the test sensibility. Horizontal walking is better to discriminate lesion-related motor deficits in the forelimb, whereas downward walking demonstrates hind limb use most sensitively.

The main study’s limit is not evaluating the anxiety and stress (main for the necessity of immobilization and some stressful triggers to perform the tests) of the animals that could affect the results ^33^ , mainly the number of times which is necessary to perform one behavioral or functional assessment test. Another limit is that some other important tests could be excluded because we cannot do or due to, we don’t assert in the review of the literature performed by the authors.

## Conclusions

The behavioral and functional assessment of peripheral nerve regeneration are low-cost, easy to performed and reliable tests. However, they need to be performed by experienced researchers to avoid misinterpretation. In brachial plexus studies, the best side to analysis is the right-side and in sciatic nerve, studies are the left side.
